# Determinants of health, health behaviours and demographic profile of patients attending an Australian university student-led osteopathy clinic

**DOI:** 10.1186/s12998-019-0292-5

**Published:** 2020-01-21

**Authors:** Brett Vaughan, Kylie Fitzgerald, Michael Fleischmann, Jane Mulcahy

**Affiliations:** 1 Department of Medical Education, University of Melbourne, Melbourne, Australia; 2 Independent researcher, Melbourne, Australia; 3 College of Health and Biomedicine, Victoria University, Melbourne, Australia

**Keywords:** Musculoskeletal, Osteopathic medicine, Health literacy, Public health, Chronic disease, Life satisfaction, General health, Spine

## Abstract

**Background:**

Profiles of health professions practice can inform pre-professional education, provide evidence to assist with interprofessional practice, and inform policy development. An understanding of the profile of patients seeking osteopathy care is emerging. Current research suggests that musculoskeletal presentations predominate with approximately one-third of patients presenting with co-morbid diseases. There is little data on these presentations in Australian osteopathy practice. This study aimed to describe the patient demographics, clinical presentations, health behaviours and determinants of health, including health literacy, of those attending for care at an Australian student-led osteopathy clinic.

**Methods:**

A convenience sample design was utilised where consecutive patients presenting for their initial consultation were invited to complete a health information questionnaire during 2016–2017. The questionnaire explored a range of health behaviours and the patient’s health status. Data from the clinical records were also extracted to establish the presenting complaint, duration of the complaint and pertinent demographics. Descriptive statistics were generated for each variable.

**Results:**

Data were available for 1617 patients presenting for their initial consultation. The mean age of patients was 33.7 (±13.1) years with 55% (*n* = 887) identifying as female. Acute presentations predominated (*n* = 840, 52%), with presentations affecting the spine being the most common (57.8%). Most patients rated their health status as *good* to *very good* (75%). Approximately 7.5% of patients were identified as having low health literacy and 55.9% were currently suffering from one or more co-morbid presentations.

**Conclusions:**

The demographic profile and presenting complaints of patients presenting to a student-led osteopathy clinic are largely consistent with other Australian private practice profiles. The current work also identified co-morbid presentations, and positive and negative health behaviours. Osteopaths may play a role in the management of, or referral for, these presentations where health behaviours require change, or management of co-morbid conditions is beyond the scope of practice. The increasing volume of patient profile literature globally suggests that osteopaths can play a substantial role in the management of musculoskeletal complaints. Further, osteopathy may play a role in screening determinants of health, and engage in multidisciplinary care to ensure those patients with co-morbid conditions or adverse health behaviours are managed appropriately.

## Background

Australian osteopaths are primary contact health professionals who are likely to encounter patients presenting with co-morbid chronic diseases [1]. Chronic diseases are considered to be those that are long lasting and have persistent effects [2]. The Australian Institute of Health & Welfare (AIHW) commonly reports on 8 major chronic diseases: arthritis, asthma, back pain, cancer, cardiovascular disease, chronic obstructive pulmonary disease, diabetes and mental health conditions [3]. These chronic diseases are becoming increasingly common in the Australian population, with cancer, coronary heart disease and diabetes reported as the leading cause of morbidity and mortality in Australia [2]. Further, there is evidence to support a relationship between these diseases and musculoskeletal complaints. Literature reports up to a 17% increase in the risk of developing a chronic disease compared to people without a musculoskeletal condition [4].

It is universally accepted that the determinants of health include individual or societal factors that influence a person’s health [5]. They include social determinants such as gender, ethnicity, education, place of residence, and employment. These social determinants are considered to influence health more broadly [3]. Biomedical determinants such as high blood pressure, high blood lipid levels and high/poorly controlled blood glucose can all influence health. Further, health behaviours such as smoking, alcohol consumption, poor nutrition and physical inactivity can have a detrimental effect on health, these behaviours may be evaluated within the osteopathy consultation. Recording determinants of health as part of a screening procedure in osteopathy clinics may identify patients with modifiable risk behaviours. There is currently little research on the prevalence of these determinants in Australian osteopathy practice.

The term *health literacy* refers to a set of skills that people need to function effectively in the health care environment [6]. In the private practice context health literacy would include the ability to read and interpret text; use quantitative information for tasks; adhere to exercise prescription regimens; and speak and listen effectively. However, our knowledge of the impact of this construct in younger populations and in populations with primary musculoskeletal complaints is limited [7–9]. Relationships between treatment outcomes and health literacy are also inconsistent [10]. Additionally, satisfaction with life may also play a role in an individuals’ health status. Satisfaction with life (SWL) is a construct thought to capture how people feel about their life in general and is not related to how they currently feel, or how satisfied they are with a specific aspect of their life [11–14].

Osteopaths registered in Australia are primary-contact health professionals. The majority of patient’s access osteopathy care privately and do not require a referral from another health professional. That said, Burke et al. [1] identified that one-in-six patients were referred to an osteopath by another health professional. With respect to interventions provided by osteopaths, previous work suggests that osteopaths use a range of manual therapy techniques in addition to exercise prescription and education (i.e. nutrition advice, stress management) in the management of their patients [1, 15, 16].

There is an emerging literature on the patient profile of those who seek osteopathy care internationally [17–20]. However, what is known about the profile of those patients seeking care from an osteopath in Australia is limited to work in 2009 by Orrock [21] and 2013 by Burke et al. [1]. More recent workforce data [15] has also contributed to our understanding of who the Australian osteopath is and has provided more detail about the patient cohort seeking osteopathy care. These practice profiles utilised different methodologies, however all three studies suggested that patients sought care for complaints predominantly affecting the cervical and lumbar spine. Burke et al. [1] also identified that over one-third of patient’s seeking osteopathy care also present with one or more co-morbid chronic diseases and this appears to be consistent with international practice profiles [16, 19]. These studies reported characteristics of osteopathic patients in private practice settings. There is little data available from student-led clinical settings. The objective of this study was to describe the patient demographics, clinical presentations, determinants of health, health behaviours and health status of those attending for care at an Australian student-led osteopathy clinic.

## Method

The study was approved by the Victoria University (VU) Human Research Ethics Committee (15–003).

### Location

The study was undertaken in the VU Osteopathy Clinic, a student-led teaching clinic at Victoria University (Melbourne, Australia). Osteopathy students complete their clinical placements in years 3, 4 and 5 of the teaching programs. There were three clinic locations at the time of the study: one in the Melbourne central business district; and two in the western suburbs of Melbourne. At these clinics, student osteopaths provide osteopathic management to members of the public, under the supervision of registered osteopaths with no referral required.

### Participants

All patients presenting to the VU Osteopathy Clinic for their initial consultation, were invited to complete the health information form in the waiting area prior to their initial treatment. The data collection period was from February 2016 until December 2017. Responses were excluded if the patient was under the age of 18, did not complete the health information form, or declined to participate by selecting this option on the form. There were no additional inclusion criteria apart from the ability to complete the written health information in English.

### Data collection

Standard practice in the clinics is that prior to their initial consultation, each patient is required to complete a personal information questionnaire and health privacy consent form. To capture data consistent with the research aims, an additional health information questionnaire was also included, and patients could choose whether to complete this questionnaire or not. Non-completion did not impact their ability to receive treatment at the clinic.

The health information questionnaire asked questions about a range of demographic and health information. Each of the items is described in Table 1 and an example form provided at Additional file 1. Minor modifications to the health information form were made between 2016 and 2017, to capture additional health and demographic data, based on research and population studies that became available during the study period [3].

**Table 1:** Social and behavioural determinants of health items on the new patient form

Item	Response	Explanation	Reference/s
Social determinants			
In which country were you born?	Free text response.	Related to health literacy and understanding of the Australian health system.	[22]
Do you speak English at home?	Yes/no.	Linked with health literacy	[8, 22]
Do you live alone?	Yes/no.	Linked with health literacy and social support	[22]
Do you have private health insurance?	Yes/no.	Linked with health literacy and health system access	[22]
Do you have a healthcare card?	Yes/no.	Linked with health literacy and health system access	[22]
Are you confident completing medical forms?	Not at all confident to Extremely confident.	Health literacy screening item	[23]
What is the highest level of education you have completed?	Primary school or less to University.	Linked with health literacy	[8, 22]
Behavioural determinants			
Do you smoke?	Yes/no.	Linked with health literacy and increased incidence of other non-communicable chronic diseases	[22]
How many hours of sleep do you get each night?	Less than 6 h to 9 or more hours.	Related to chronic musculoskeletal complaints	
How many serves of fruit do you consume each day?	0 to 7.	Linked with health literacy and general health behaviours. Evaluated in Australian National Health Survey	[24]
How many serves of vegetables do you consume each day?		Linked with health literacy and general health behaviours. Evaluated in Australian National Health Survey	[24]
Over the last week, how many days did you exercise for at least 30 min per day?	0 to 7.	Establish health behaviours in relation to Australian guidelines	
How long would each typical exercise session last?^a^	Less than 30 min to Greater than 90 min.	Establish health behaviours in relation to Australian guidelines	
On average, rate the intensity of your exercise over the last week?^a^	Sedentary to High.	Establish health behaviours in relation to Australian guidelines	
On a usual week day, how much time do you spend sitting: - As part of work or volunteer activities? In other leisure time?	0–3 h to 12 h or more.	Linked with health literacy and general health behaviours. Evaluated in Australian National Health Survey	[24]
Have you had your blood pressure checked by a doctor or health professional in the past 12 months?	Yes/no.	Linked with health literacy and general health behaviours.	
Have you had a skin cancer check in the past 12 months?	Yes/no.	Common issue in Australia and linked with general health behaviours.	
Chronic disease status			
Common chronic diseases: arthritis, back pain, asthma, heart problems, high cholesterol, high blood pressure, asthma, cancer, mental health disorder, stroke, diabetes, kidney disease	Currently suffering/Previously suffering/Both.	Common chronic diseases evaluated in the Australian National Health Survey. The list on the form varied from 2016 to 2017 due to a change in the prevalence of some chronic diseases relative to others	[2, 3]
Global health status			
Please rate your general health	Poor to Excellent.	Evaluated in Australian National Health Survey.	[24]
Quality of life			
Overall, how satisfied are you with your life?	0 (not at all satisfied) to 5 (extremely satisfied).	Potential screen for a range of mental health disorders and health literacy.	[8]

^a^ Only completed by patients attending the clinic in 2016

Additional demographic and clinical information was extracted from the patients’ electronic health record by one researcher (BV) then de-identified. Data extracted included occupation, postcode, gender, region of the presenting complaint, diagnosis coded using the International Classification of Disease (ICD-10), chronicity of the complaint (acute/chronic), age, and blood pressure (where recorded in the health record). Patient postcode was classified according to the Socio-Economic Indexes for Areas (SEIFA) Index of Relative Socio-economic Advantage and Disadvantage [25] and occupation was coded according to the Australian Bureau of Statistics [26].

### Data analysis

All data were deidentified by the lead author (BV) prior to entry into SPSS (IBM Corp, USA). Missing data were not imputed. Descriptive statistics were generated for each of the demographic and health information items.

## Results

One thousand eight hundred forty initial consultations were undertaken during the data collection period. Data from 1614 patients was available for analysis representing an 87.9% response rate. Where data was not available this was due to patients declining to have their data included in the analysis, the health information questionnaire was not completed or were under the age of 18 years. The split of data between years was approximately even (2016–49.9%, 2017–50.1%) with 83.2% (*n* = 1338) attending the Melbourne CBD clinic and the remainder attending the clinics in the western suburbs of Melbourne (Werribee and St Albans).

### Patient clinical and demographic profile

**Fig. 1:**
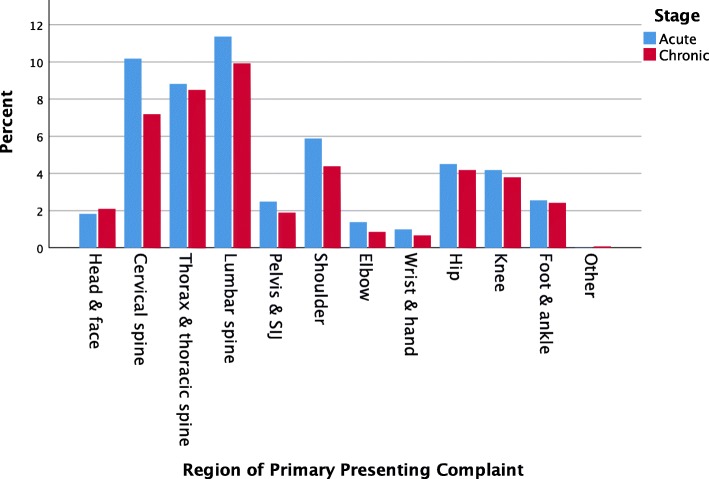
Region of primary diagnosis of patients presenting to the Victoria University Osteopathy Clinic in 2016–2017

Demographic data for the patient population are detailed in Table 2. Osteopathy students accounted for 5.1% (*n* = 82) of the patient cohort. The majority of patients were employed with 3.3% (*n* = 54) unemployed, 3.0% (*n* = 49) retired and 1.2% (*n* = 19) undertaking home duties. Just over half of the patients presented with a complaint of less than 3 months duration (*n* = 840, 52%), with the most common presentations being those affecting the lumbar spine (21.2%) (Fig. 1). Acute presentations were more likely to be associated with lumbar (21%) and cervical spine (18.8%) complaints. Chronic presentations were more likely to be thoracic spine/thorax (18.5%) and lumbar spine (21.6%). The International Classification of Disease version 10 (ICD-10) was used to code each diagnosis (Additional file 2). These classifications demonstrate the breadth of diagnoses based on patient presentations to the clinic.

**Table 2:** Descriptive statistics for the demographics of patients presenting to the Victoria University Osteopathy Clinic in 2016–2017

Gender	
Male	675 (41.9%)
Female	887 (54.9%)
Missing	52 (3.2%)
Age	
Mean (±SD)	33.7 (±13.1) years
Median	29 years
Range	18–85 years
Stage	
Acute	840 (52.0%)
Chronic	711 (41.1%)
Missing	63 (3.9%)
SEIFA classification	
0-5th decile	380 (23.5%)
6th–7th decile	314 (19.4%)
8th–9th decile	621 (38.5%)
10th decile	269 (16.7%)
Missing	30 (1.9%)

*SEIFA* Socio-Economic Indexes for Areas (1st decile = lowest socioeconomic area, 10th decile = highest socioeconomic area)

### Social and behavioural determinants of health

**Fig. 2:**
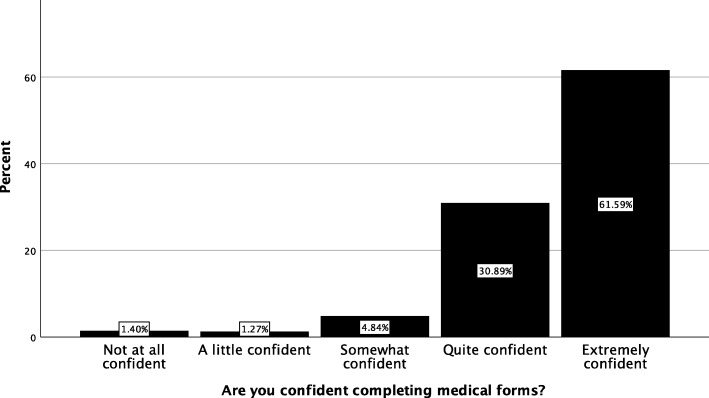
Self-rated health literacy using a single-item screening question for patients presenting to the Victoria University Osteopathy Clinic in 2016–2017

Descriptive data for the social and behavioural determinants of health are presented in Table 3. Health literacy was screened by way of a single item and the results displayed in Fig. 2. One-hundred and eighteen patients (7.5%) reported feeling *somewhat confident* or less suggesting these patients may have limited health literacy.

**Table 3:** Descriptive statistics for the social and behavioural determinants of health of patients presenting to the Victoria University Osteopathy Clinic in 2016–2017

Social determinants	
In which country were you born?	
Australia	1023 (63.4%)
Other	591 (36.6%)
Missing	0
Do you speak English at home?	
Yes	1443 (89.4%)
No	160 (9.9%)
Missing	11 (0.7%)
Do you live alone?	
Yes	127 (7.9%)
No	722 (44.8%)
Missing	765 (47.4%)
Do you have private health insurance?	
Yes	378 (23.4%)
No	493 (30.5%)
Missing	743 (46.0%)
Do you have a healthcare card?	
Yes	363 (22.5%)
No	500 (31.0%)
Missing	751 (46.5%)
What is the highest level of education you have attended?	
Primary school or less	9 (0.6%)
High school (not completed)	65 (4%)
High school (completed)	264 (16.4%)
TAFE or trade qualification	251 (15.6%)
University	1006 (62.3%)
Missing	19 (1.1%)
Behavioural determinants	
Do you smoke?	
Yes	196 (12.1%)
No	1297 (80.4%)
Missing	121 (7.5%)
How many hours of sleep do you get each night?	
Less than 6 h	143 (8.9%)
7–8 h	853 (52.9%)
9–10 h	541 (33.5%)
Greater than 10 h	59 (3.7%)
Missing	18 (1.1%)
How many serves of fruit do you consume each day? (median, range)	2 serves (range 0–8) Missing: 20 (1.2%)
How many serves of vegetables do you consume each day? (median, range)	3 serves (range 0–8) Missing: 19 (1.1%)
How many exercise sessions would you do each week? (median, range)	3 sessions (range 0–8) Missing: 35 (2.2%)
Have you had your blood pressure checked in the last 6 months?	
Yes	962 (59.6%)
No	601 (37.2%)
Missing	51 (3.1%)
Have you had a skin cancer check in the last 6 months?	
Yes	113 (7%)
No	762 (47.2%)
Missing	739 (45.8%)

**Fig. 3:**
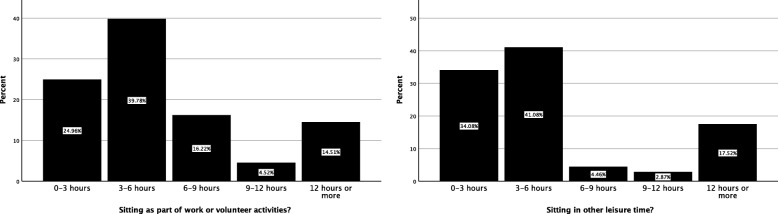
Sitting as part of work/volunteer activities and in other leisure activities for patients presenting to the Victoria University Osteopathy Clinic in 2016–2017

Sitting related to employment/volunteering, or as a part of leisure activities was also evaluated, with 3–6 h per day being most common for both scenarios (Fig. 3). Most patients (86.4%) reported between 7 and 10 h of sleep per evening. The median number of exercise sessions per week was three, with 6.2% (*n* = 98) not undertaking any exercise. For those patients who indicated exercising, the average duration of each exercise session was between 30 and 60 min, with most undertaking low (297, 18.4%) to medium intensity (*n* = 312, 19.3%) exercise.

### Chronic conditions

Patients were asked to indicate if they had a current major chronic illness or had previously experienced one of the major chronic diseases affecting the Australian population [24, 27]. Back pain (42.6%) and mental health disorders (8.6%) were the most common conditions affecting the patient population on presentation to the clinic (Table 4).

**Table 4:** Common chronic conditions experienced by patients presenting to the Victoria University Osteopathy Clinic in 2016–2017

Chronic condition	Current history	Previous history	Both current & previous	Australian National Health Survey [24]
Arthritis	105 (6.6%)	33 (2%)	3 (0.2%)	15.3%
Back pain	372 (42.6%)	116 (13.3%)	67 (7.7%)	16%
Heart problems	33 (2.1%)	38 (2.4%)	1 (0.1%)	5.2%
High blood pressure/cholesterol^a^	49 (5.6%)	41 (4.7%)	5 (0.6%)	
High cholesterol	27 (3.7%)	23 (3.2%	1 (0.1%)	7.1%
High blood pressure	24 (3.3%)	25 (3.5%)	2 (0.3%)	11.3%
Asthma	118 (7.4%)	179 (11.2%)	5 (0.3%)	10.8%
Cancer	14 (0.9%)	45 (2.8%)	1 (0.1%)	1.6%
Mental health disorder	137 (8.6%)	143 (8.9%)	21 (1.3%)	17.5%
Diabetes	18 (1.1%)	28 (1.8%)	1 (0.1%)	5.1%
Stroke	2 (0.2%)	8 (0.9%)	0	2%
Kidney disease	2 (0.3%)	11 (1.6%	0	0.9%

^a^ question was combined in the 2016 patient information questionnaire

### Global health status and quality of life

**Fig. 4:**
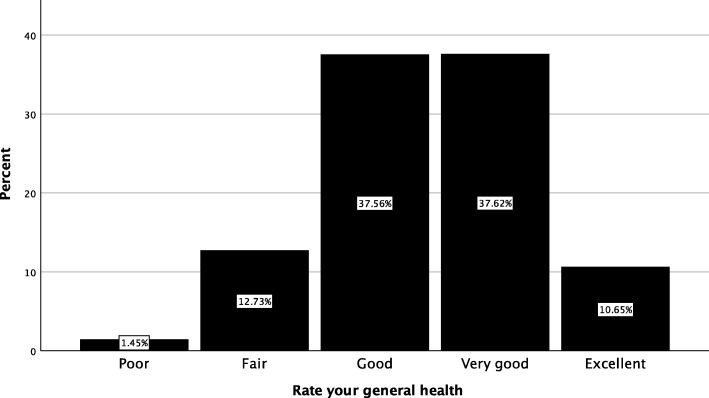
Self-rated general of patients presenting to the Victoria University Osteopathy Clinic in 2016–2017

**Fig. 5:**
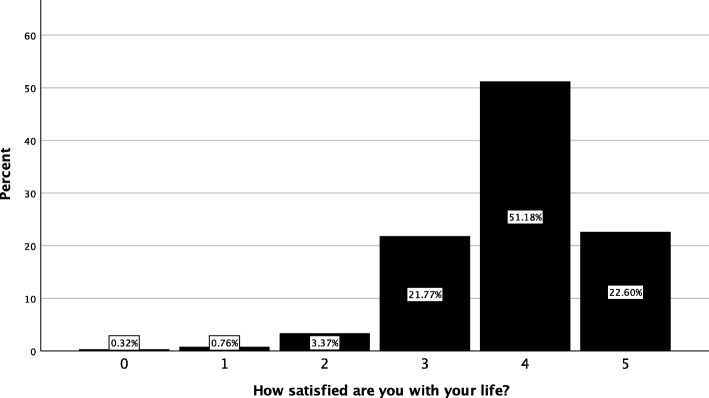
Satisfaction with life of patients presenting to the Victoria University Osteopathy Clinic in 2016–2017

Global health status was evaluate using two items and descriptive statistics are provided in Table 5. The median general health status rating was good (3) (Table 5) with a range of *poor* to *excellent* (Fig. 4) and median life satisfaction rating was 4 (Fig. 5).

**Table 5:** Descriptive statistics for the global health status of patients presenting to the Victoria University Osteopathy Clinic in 2016–2017

Please rate your general health	
Median	3 (good)
Range	1 (poor) – 5 (excellent)
Quartiles	25%: 3, 50%: 3, 75%: 4
Missing	27 (1.7%)
How satisfied are you with your life?	
Median	4
Range	0–5
Quartiles	25%: 3, 50%: 4, 75%: 4
Missing	43 (2.7%)

## Discussion

This study contributes to an increased understanding of the demographic and clinical profile, conditions, health status and health determinants, of patients seeking osteopathy care from student osteopaths in Australia. As highlighted, previous research of Australian osteopathy practice has used limited data collection periods [1, 21], or practitioner-reported perception of the patient profile of their practice [15]. The current work builds on these studies by utilising consecutive patient data over a 22-month period from a student-led osteopathy clinic. This data therefore adds another dimension to our understanding of this patient profile and contributes to the international discourse with respect to osteopathic patient characteristics, conditions and behaviours [19]. As Australian osteopathy students are trained as primary care professionals, they are well placed to screen and manage some aspects of the patients’ care, to refer when appropriate to do so, or participate in multidisciplinary care.

### Patient clinical and demographic profile

The patient demographic profile from this student-led osteopathy clinic were largely consistent with Australian private osteopathy practice data [1, 15, 21]. Differences include a lower percentage of females (52%), lower mean age, a higher percentage born overseas and higher number of non-English speaking patients. As a student led clinic offers a reduced rate for consultations, it is probable that there may be some differences in the patient demographics.

The education level of patients attending for osteopathy care in Australia has not previously been reported. The current study provides initial evidence to suggest that the majority of patients seeking osteopathy care have attended higher education. However, this may be a product of clinic location (central business district) where the majority of patients attended, and the clinic being located at a university. Higher levels of education have been associated with higher health literacy and potentially an awareness of the role of osteopathy in the health system [8].

Spinal complaints comprised the majority of acute and chronic presentations (42.6%) with lumbar spine complaints being the most common. Specifically, cervicalgia (M54.2) and Strain/Sprain of the lumbar spine (S33.5) were the most common diagnoses using the ICD-10 classification (Additional file 1). The use of this diagnostic system on a consistent basis across the profession, may help to provide a more accurate picture of the breadth of conditions treated by Australian osteopaths [28]. This finding is consistent with the literature suggesting that the management of spinal complaints forms a core aspect of the practice of osteopathy in Australia and internationally [1, 15, 16, 18–21]. More patients presented with acute presentations of less than 3 months duration (52%) than chronic presentations. Previous studies have reported lower rates of acute presentations (45.1% [19] and 48.2% [1]) where acute was defined as less than 4 weeks duration. Therefore, it is difficult to compare this data due to these temporal definition differences.

There was a small subset of patients (7.5%) with limited health literacy attending the student clinic, consistent with Vaughan et al. [8]. This outcome is less than Australian population data and may be higher in the population seeking care at the teaching clinic. This is plausible, considering the higher level of education of the current clinical cohort (university 62.2%). This level of education for those aged 30–39 years is consistent with the Australian population [29].

### Behavioural determinants of health

Just over 12% of patients indicated they were smoking at the time of completing the questionnaire. Consecutive Australian National Health surveys have indicated that smoking rates are decreasing with most recent data suggesting that 14.5% of adults are smoking [24]. Lower rates in the present study may be due to the predominately younger population in the present study, as smoking rates have dropped significantly in those aged 18–44 years 22.8% in 2001 to 16.3% in 2014–2015 [24].

Patient self-report data suggested that median fruit and vegetable consumption is consistent with Australian population health data [30], but lower than the Australian guidelines [31]. Approximately half of the patients in the present study reported exercising at levels that met guidelines consistent with Australian National Health Survey data [24]. These outcomes provide osteopaths with an opportunity to play a role in the education of patients about healthy eating and exercise consistent with public health guidelines, given the strong associations of these behaviours with chronic illnesses [2] and musculoskeletal health [32].

Sitting is increasingly considered to be a factor influencing musculoskeletal health, in particular low back pain [33] and neck-shoulder pain [34]. Most patients in the present study reported sitting up to 12 h per day across work and leisure activities. Extrapolating the current work to hours per week, would see patients seated for approximately 30 h per week during occupational activities and a similar volume with leisure activities. The former result is somewhat less than the Australian National Survey data [35] and this may be due to the increasing usage of ergonomic devices such as stand-up desks [36]. Changes with respect to sedentary behaviours in the workplace may also lead to reductions in musculoskeletal complaints [37].

Poor or limited sleep is recognised as a potential contributor to a range of chronic conditions, including musculoskeletal complaints [38, 39]. The majority of patients (52.9%) self-reported having 7–8 h of sleep per night and this is consistent with work by Adams et al. [40] in an Australian community sample. The questionnaire in the current study did not address sleep quality, or difficulty with sleep, therefore patients may have sleep issues that are not addressed here [40].

### Chronic disease

Presence of co-morbid chronic conditions alongside musculoskeletal complaints is common [41] and has been reported in patients presenting to Australian osteopaths [1]. These studies highlight the need to improve our understanding of the prevalence of these diseases in our patient population, as addressing musculoskeletal complaints may reduce the burden of chronic disease [4]. Overall, the presence of chronic diseases in the patient cohort was typically less than the Australian population, a result likely due to the younger population in the current work.

Mental health complaints are reported to be experienced by 17.5% of the population [24] however, the patient cohort reported rates of 8.9% currently experiencing a mental health issue was lower. When combining the reported current, past, and current/past history of mental health ‘a history of’ mental health complaints’, the data (18.7%) is relatively consistent with the Australian population (17.5%) [24]. There may an unwillingness to self-report having or experiencing a mental health issue, or having received a mental health diagnosis This may account for patients underreporting of ‘currently experiencing’ a mental health disorder. As such, it may be valuable to explore other strategies to screen for mental health disorders so that patients can receive the appropriate care. The prevalence of mental health disorders is likely underreported in the current study.

### Global health status

Self-rated general health in the patient cohort was lower than for the Australian population described in the Australian National Health Survey [24]. Rates between the Australian population and the current cohort were consistent for the *very good* (37%) and *poor/fair* ratings (15%). The *excellent* rating was lower in the current study (10% v 20%) but higher for the *goo*d rating (37% v 29%) [24]. It is not clear why these differences were observed as the same item was used in the current study and the National Health Survey [24]. However, it is posited that as the patients were seeking a health service at the time of completing the question, this may account for the lower rates of higher self-reported general health status.

### Quality of life

The majority of the cohort (70%) rated their satisfaction with life at 4 or 5 (out of 5), suggesting they were largely satisfied with their life. Although consistent with Australian data, the current cohort was relatively well educated, and this may be a factor leading to greater life satisfaction [42]. The evaluation of life satisfaction in osteopathy patients is valuable, given the reported associations with self-reported general health, the absence of chronic conditions, and participating in positive health behaviours across the Australian population [43]. Further, positive health behaviours (e.g. exercising, not smoking, fruit/vegetable consumption) have been associated with self-reported life satisfaction [44].

### Limitations

There are a number of limitations to the current work that may limit its generalisability. Firstly, the self-report nature of the study requires acknowledgment and objective measures of a number of aspects of the study would be required to confirm patient responses. The health information and demographic questionnaires were designed to ensure they could be completed in a timely manner by the patient prior to their consultation, while not being burdensome. Further, patients may choose to not answer some questions on the questionnaire, and this may distort the true patient profile. This was evident for demographic items and health behaviours. The use of single item questions requires further exploration and analysis. To be validated these single items need to be administered along with other measures that have been found to be valid and reliable, multi-item measures of the same construct. Some of this work is currently underway in the institution. Another limitation was that each item on the questionnaire, apart from “In which country were you born?”, had missing data in the present study however this is unlikely to significantly influence the description provided here.

The data was obtained from one clinical environment, a student-led teaching clinic. Although the teaching environment may be different to the private clinical practice environment where most Australian osteopaths work [15] thereby limiting the generalisability. As the work is specific to the Australian context, its generalisability to international practice profiles may also be limited. Our understanding of health behaviours and demographics is constantly evolving, therefore the use of a number of items that measure these constructs on the new patient clinic form, used in the present study may be redundant and require modifications in the future.

## Conclusion

This study has provided a clinical and demographic patient profile derived from the clinical history taken by student osteopathy practitioners and self-report patient data in a student-led osteopathy clinic. This patient profile is largely consistent with previously published Australian clinical practice profiles. This result has clinical practice, research and educational implications. From a clinical practice standpoint, we now have an increased understanding of the social and behavioural determinants of health, and demographics of patients seeking osteopathy care, particularly in the student-led clinical teaching environment. With respect to future research, the data presented here provides researchers, educators and those with an interest in health policy a greater level of detail about the patient seeking osteopathy care in this setting. From an educational standpoint, this data can be used to ensure that curricula address the range of musculoskeletal presentations, health status, health behaviours and quality of life of patients presenting for osteopathic care at a student-led clinic.

## Supplementary information

**Additional file 1.** Health Information Questions

**Additional file 2.** ICD-10 Coding.

## Data Availability

The datasets used and/or analysed during the current study are available from the corresponding author on request.

## Abbreviations

ICD: International Classification of Disease
SEIFA: Socio-Economic Indexes for Areas
