# Supplementary material for: Determinants of health, health behaviours and demographic profile of patients attending an Australian university student-led osteopathy clinic
Source: Chiropr Man Therap. 2020 Jan 21;28:2. doi: 10.1186/s12998-019-0292-5 (PMC6971880; doi:10.1186/s12998-019-0292-5)
Supplement: Supplementary file 1 — Additional file 1. Health Information Questions [file 12998_2019_292_MOESM1_ESM.pdf]

Health Information Questions

To assist us with providing quality care, monitor treatment outcomes, and improve the service provided at the clinic, we would appreciate you taking the time to answer the following questions. The information that you provide here may also be used for research purposes, and where this occurs no information that could identify you will be used, analysed or published. You may also choose not to have this information made available to researchers in the Victoria University osteopathy course.

In which country were you born? ____

Do you speak English at home? ☐ Yes ☐ No **Do you smoke?** ☐ Yes ☐ No

How often do you have someone help you read hospital materials? ☐ Always ☐ Most times ☐ Sometimes ☐ Rarely ☐ Never

How often do you have problems learning about your medical condition because of difficulty understanding written information? ☐ Always ☐ Most times ☐ Sometimes ☐ Rarely ☐ Never

Are you confident completing medical forms? ☐ Not at all confident ☐ A little confident ☐ Somewhat confident ☐ Quite confident ☐ Extremely confident

How often do you have a problem understanding what is told to you about your medical condition? ☐ Always ☐ Most times ☐ Sometimes ☐ Rarely ☐ Never

What is the highest level of education you have completed?
☐ Primary school or less ☐ Some high school ☐ High school ☐ TAFE or trade qualification ☐ University

How many hours of sleep do you get each night? ☐ Less than 6 hours ☐ 6-7 hours ☐ 7-8 hours ☐ 9 or more hours

How many serves of fruit do you consume each day? (please circle) 0 1 2 3 4 5 6 7

How many serves of vegetables do you consume each day? (please circle) 0 1 2 3 4 5 6 7

Over the last week, how many days did you exercise for at least 30 minutes per day? 0 1 2 3 4 5 6 7

On a usual week day, how much time do you spend sitting:

As part of work or volunteer activities? ☐ 0-3 hours ☐ 3-6 hours ☐ 6-9 hours ☐ 9-12 hours ☐ 12 hours or more

In other leisure time? ☐ 0-3 hours ☐ 3-6 hours ☐ 6-9 hours ☐ 9-12 hours ☐ 12 hours or more

Have you had your blood pressure checked by a doctor or health professional in the past 12 months? ☐ Yes ☐ No

Do you have or ever suffered from any of the following? (please tick all that apply)

	**Currently suffering**	**Previously suffered**
Arthritis	☐	☐
Heart problems	☐	☐
High cholesterol	☐	☐
High blood pressure	☐	☐
Asthma	☐	☐
Cancer	☐	☐
Mental health disorder (i.e. depression, anxiety)	☐	☐
Diabetes	☐	☐
Kidney disease	☐	☐

Please rate your general health: ☐ Poor ☐ Fair ☐ Good ☐ Very good ☐ Excellent

Overall, how satisfied are you with your life? (please circle) (not at all satisfied) 0 1 2 3 4 5 (extremely satisfied)
