# Supplementary material for: Determinants of health, health behaviours and demographic profile of patients attending an Australian university student-led osteopathy clinic
Source: Chiropr Man Therap. 2020 Jan 21;28:2. doi: 10.1186/s12998-019-0292-5 (PMC6971880; doi:10.1186/s12998-019-0292-5)
Supplement: Supplementary file 2 — Additional file 2. ICD-10 Coding. [file 12998_2019_292_MOESM2_ESM.pdf]

ICD10

		Frequency	Percent	Valid Percent	Cumulative Percent
Valid		66	4.1	4.1	4.1
	G43.0 Migraine without aura [common migraine]	2	.1	.1	4.2
	G43.1 Migraine with aura [classical migraine]	1	.1	.1	4.3
	G44.2 Tension-type headache	8	.5	.5	4.8
	G44.8 Other specified headache syndromes (cervicogenic, refractive error, sinus, TMJ)	35	2.2	2.2	6.9
	G54.0 Thoracic outlet syndrome	12	.7	.7	7.7
	G56.0 Carpal tunnel syndrome	2	.1	.1	7.8
	G56.1 Other lesions of median nerve	1	.1	.1	7.9
	Other mononeuropathy of upper limb	1	.1	.1	7.9
	G57.0 Lesion of the sciatic nerve	1	.1	.1	8.0
	G57.1 Meralgia paraesthetica	1	.1	.1	8.1
	Mortons neuroma	1	.1	.1	8.1
	K07.6 Temporomandibular joint disorders (chronic)	14	.9	.9	9.0
	M10.0 Gout	1	.1	.1	9.0
	M16.0 Primary coxarthrosis (bilateral hip OA)	1	.1	.1	9.1
	M16.1 Other primary coxarthrosis (unilateral hip OA)	3	.2	.2	9.3
	M17.0 Primary gonarthrosis (bilateral knee OA)	1	.1	.1	9.4
	M17.1 Other primary gonarthrosis (unilateral knee OA)	1	.1	.1	9.4
	M17.2 Post-traumatic gonarthrosis, bilateral (knee)	1	.1	.1	9.5
	M17.3 Post-traumatic gonarthrosis	1	.1	.1	9.5
	M18.1 Other primary arthrosis of first carpometacarpal joint	1	.1	.1	9.6

ICD10

	Frequency	Percent	Valid Percent	Cumulative Percent
M19.0 Primary arthrosis of other joints	1	.1	.1	9.7
M19.1 Post-traumatic arthrosis of other joints	1	.1	.1	9.7
M22.2 Patellofemoral disorders	30	1.9	1.9	11.6
M22.4 Chondromalacia patellae	3	.2	.2	11.8
M23.2 Derangement of meniscus due to old tear or injury (Old bucket-handle tear)	2	.1	.1	11.9
M23.3 Other meniscus derangements	7	.4	.4	12.3
M25.3 Other instability of joint	1	.1	.1	12.4
M25.4 Effusion of joint	1	.1	.1	12.5
M25.8 Other specified joint disorders (FAI, ligamentum teres)	28	1.7	1.7	14.2
M43.1 Spondylolithesis	1	.1	.1	14.3
M45.0 Ankylosing spondylitis	1	.1	.1	14.3
M46.8 Other specified inflammatory spondylopathies	1	.1	.1	14.4
M47.8 Spondylosis without myelopathy or radiculopathy	19	1.2	1.2	15.6
M50.1 Cervical disc disorder with radiculopathy	2	.1	.1	15.7
M50.3 Other cervical disc degeneration	1	.1	.1	15.7
M50.9 Cervical disc disorder, unspecified	5	.3	.3	16.0
M51.1 Lumbar and other intervertebral disc disorders with radiculopathy	11	.7	.7	16.7
M51.9 Intervertebral disc disorder, unspecified	64	4.0	4.0	20.7
M53.3 Sacrococcygeal disorders (coccygodynia)	5	.3	.3	21.0
M54.2 Cervicalgia	148	9.2	9.2	30.2
M54.3 Sciatica	1	.1	.1	30.2

ICD10

	Frequency	Percent	Valid Percent	Cumulative Percent
M54.4 Lumbago with sciatica	1	.1	.1	30.3
M54.5 Low back pain	95	5.9	5.9	36.2
M54.6 Pain in thoracic spine	97	6.0	6.0	42.2
M65.3 Trigger finger	1	.1	.1	42.3
M65.4 Radial styloid tenosynovitis [de Quervain]	2	.1	.1	42.4
M70.2 Olecranon bursitis	2	.1	.1	42.5
M70.4 Prepatella bursitis	1	.1	.1	42.6
M70.5 Other bursitis of the knee	3	.2	.2	42.8
M70.6 Trochanteric bursitis	6	.4	.4	43.1
M72.2 Plantar fasciopathy	11	.7	.7	43.8
M72.8 Traumatic plantar fasciopathy	1	.1	.1	43.9
M75.0 Adhesive capsulitis of shoulder	2	.1	.1	44.0
M75.1 Rotator cuff syndrome	20	1.2	1.2	45.2
M75.2 Bicipital tendinitis	24	1.5	1.5	46.7
M75.4 Impingement syndrome of shoulder	25	1.5	1.5	48.3
M75.5 Bursitis of shoulder	7	.4	.4	48.7
M75.8 Other shoulder lesions	1	.1	.1	48.8
M76.0 Gluteal tendinitis	17	1.1	1.1	49.8
M76.3 Iliotibial band syndrome	6	.4	.4	50.2
M76.5 Patella bursitis	5	.3	.3	50.5
M76.6 Achilles tendinitis	8	.5	.5	51.0
M76.7 Peroneal tendonitis	1	.1	.1	51.1
M76.8 Other enthesopathies of lower limb (Anterior tibial syndrome, Posterior tibial tendinitis)	14	.9	.9	51.9
M77.0 Medial epicondylitis	6	.4	.4	52.3

ICD10

	Frequency	Percent	Valid Percent	Cumulative Percent
M77.1 Lateral epicondylitis	15	.9	.9	53.2
M77.2 Periarthritis of wrist	1	.1	.1	53.3
M77.4 Metatarsalgia	1	.1	.1	53.3
M79.4 Fat pad pathology (knee)	2	.1	.1	53.5
M94.0 Chondrocostal junction syndrome [Tietze] (Costochondritis)	2	.1	.1	53.6
M99.9 Other musculoskeletal	1	.1	.1	53.7
Q65.8 Other congenital deformities of hip	1	.1	.1	53.7
R10.2 Pelvic pain	1	.1	.1	53.8
R51.0 Headache	1	.1	.1	53.8
S03.4 Sprain and strain of jaw (acute)	2	.1	.1	54.0
S13.4 Sprain and strain of cervical spine (Atlanto-axial (joints) Atlanto-occipital (joints) Whiplash injury)	14	.9	.9	54.8
S16.0 Injury of muscle and tendon at neck level	84	5.2	5.2	60.0
S23.3 Sprain and strain of thoracic spine	57	3.5	3.5	63.6
S23.4 Sprain and strain of ribs and sternum	92	5.7	5.7	69.3
S23.5 Sprain and strain of other and unspecified parts of thorax	1	.1	.1	69.3
S33.5 Sprain and strain of lumbar spine	104	6.4	6.4	75.8
S33.6 Sprain and strain of sacroiliac joint	58	3.6	3.6	79.4
S39.0 Injury of muscle and tendon of abdomen, lower back and pelvis	50	3.1	3.1	82.5
S43.4 Sprain and strain of shoulder joint (Coracohumeral (ligament), Rotator cuff capsule)	14	.9	.9	83.3

ICD10

	Frequency	Percent	Valid Percent	Cumulative Percent
S43.5 Sprain and strain of acromioclavicular joint (Acromioclavicular ligament)	11	.7	.7	84.0
S44.8 Injury of other nerves at shoulder and upper arm level	4	.2	.2	84.3
S46.0 Injury of muscle(s) and tendon(s) of the rotator cuff of shoulder	22	1.4	1.4	85.6
S46.1 Injury of muscle and tendon of long head of biceps	1	.1	.1	85.7
S46.2 Injury of muscle and tendon of other parts of biceps	3	.2	.2	85.9
S46.3 Injury of muscle and tendon of triceps	2	.1	.1	86.0
S46.7 Injury of multiple muscles and tendons at shoulder and upper arm level	6	.4	.4	86.4
S46.8 Injury of other muscles and tendons at shoulder and upper arm level	15	.9	.9	87.3
S53.4 Sprain and strain of elbow	1	.1	.1	87.4
S54.0 Injury of ulnar nerve at forearm level	3	.2	.2	87.5
S56.0 Injury of flexor muscle and tendon of thumb at forearm level	1	.1	.1	87.6
S56.1 Injury of long flexor muscle and tendon of other finger(s) at forearm level	6	.4	.4	88.0
S56.4 Injury of extensor muscle and tendon of other finger(s) at forearm level	2	.1	.1	88.1
S56.7 Injury of multiple muscles and tendons at forearm level	1	.1	.1	88.2
S56.8 Injury of other and unspecified muscles and tendons at forearm level	2	.1	.1	88.3
S62.0 Fracture of navicular [scaphoid] bone of hand	1	.1	.1	88.4

ICD10

	Frequency	Percent	Valid Percent	Cumulative Percent
S63.5 Strain or sprain of the wrist	10	.6	.6	89.0
S63.6 Sprain and strain of finger(s)	4	.2	.2	89.2
S66.8 Injury of other muscles and tendons at wrist and hand level	1	.1	.1	89.3
S74.0 Injury of sciatic nerve at hip and thigh level	4	.2	.2	89.5
S76.0 Injury of muscle and tendon of hip	41	2.5	2.5	92.1
S76.1 Injury of quadriceps muscle and tendon Patellar ligament (tendon)	11	.7	.7	92.8
S76.2 Injury of adductor muscle and tendon of thigh	3	.2	.2	92.9
S76.3 Injury of muscle and tendon of the posterior muscle group at thigh level	16	1.0	1.0	93.9
S76.4 Injury of other and unspecified muscles and tendons at thigh level	6	.4	.4	94.3
S76.7 Injury of multiple muscles and tendons at hip and thigh level	3	.2	.2	94.5
S82.6 Fracture of lateral malleolus	1	.1	.1	94.5
S83.2 Tear of meniscus, current	6	.4	.4	94.9
S83.4 Sprain and strain involving (fibular)(tibial) collateral ligament of knee	11	.7	.7	95.6
S83.5 Sprain and strain involving (anterior) (posterior) cruciate ligament of knee	4	.2	.2	95.8
S83.6 Sprain and strain of other and unspecified parts of knee	3	.2	.2	96.0
S83.7 Injury to multiple structures of knee	1	.1	.1	96.1
S84.0 Injury of tibial nerve at lower leg level	1	.1	.1	96.2
S86.0 Injury of Achilles tendon	1	.1	.1	96.2

ICD10

	Frequency	Percent	Valid Percent	Cumulative Percent
S86.1 Injury of other muscle(s) and tendon(s) of posterior muscle group at lower leg level	15	.9	.9	97.1
S86.2 Injury of muscle(s) and tendon(s) of anterior muscle group at lower leg level	2	.1	.1	97.3
S86.3 Injury of muscle(s) and tendon(s) of peroneal muscle group at lower leg level	5	.3	.3	97.6
S86.7 Injury of multiple muscles and tendons at lower leg level	2	.1	.1	97.7
S86.8 Injury of other muscles and tendons at lower leg level	2	.1	.1	97.8
S92.3 Fracture of metatarsal bone	2	.1	.1	98.0
S92.5 Fracture of other toe	1	.1	.1	98.0
S93.2 Rupture of ligaments at ankle and foot level	1	.1	.1	98.1
S93.4 Sprain and strain of ankle (Calcaneofibular Deltoid, Talofibular, Tibiofibular)	16	1.0	1.0	99.1
S93.5 Sprain and strain of toe(s)	2	.1	.1	99.2
S93.6 Sprain and strain of other and unspecified parts of foot	4	.2	.2	99.4
S96.0 Injury of muscle and tendon of long flexor muscle of toe at ankle and foot level	2	.1	.1	99.6
S96.1 Injury of muscle and tendon of long extensor muscle of toe at ankle and foot level	3	.2	.2	99.8
S96.2 Injury of intrinsic muscle and tendon at ankle and foot level	4	.2	.2	100.0
Total	1614	100.0	100.0	
